# Barriers to antiretroviral therapy adherence in rural Mozambique

**DOI:** 10.1186/1471-2458-11-650

**Published:** 2011-08-16

**Authors:** Kate Groh, Carolyn M Audet, Alberto Baptista, Mohsin Sidat, Alfredo Vergara, Sten H Vermund, Troy D Moon

**Affiliations:** 1 Department of Medicine, D-3100, Medical Center North, Nashville, TN 37232-2358, USA; 2 Department of Preventive Medicine, Village at Vanderbilt, Suite 2100,1500 21st Avenue South Nashville, TN 37212, USA; 3 Vanderbilt Institute for Global Health, 2525 West End Ave, Suite 750, 2525 West End Ave, Nashville, TN, 37203, USA; 4 Department of Pediatrics, 2200 Children's Way, Nashville, TN 37232, USA; 5 Ministry of Health, Av. Quelimane: Av. 1 de Julho, Predio deo Monte Giro, Quelimane, Moçambique; 6 Faculty of Medicine, University Eduardo Mondlane, PO Box 257, Maputo, Moçambique; 7 Friends in Global Health, Avenida dos Trabalhadores N°424, Quelimane, Moçambique

**Keywords:** HIV, AIDS, Mozambique, antiretroviral therapy, adherence, compliance, health care workers, attitudes, behaviors, rural, focus groups

## Abstract

**Background:**

HIV is treated as a chronic disease, but high lost-to-follow-up rates and poor adherence to medication result in higher mortality, morbidity, and viral mutation. Within 18 clinical sites in rural Zambézia Province, Mozambique, patient adherence to antiretroviral therapy has been sub-optimal.

**Methods:**

To better understand barriers to adherence, we conducted 18 community and clinic focus groups in six rural districts. We interviewed 76 women and 88 men, of whom 124 were community participants (CP; 60 women, 64 men) and 40 were health care workers (HCW; 16 women, 24 men) who provide care for those living with HIV.

**Results:**

While there was some consensus, both CP and HCW provided complementary insights. CP focus groups noted a lack of confidentiality and poor treatment by hospital staff (42% CP vs. 0% HCW), doubt as to the benefits of antiretroviral therapy (75% CP vs. 0% HCW), and sharing medications with family members (66% CP vs. 0%HCW). Men expressed a greater concern about poor treatment by HCW than women (83% men vs. 0% women). Health care workers blamed patient preference for traditional medicine (42% CP vs. 100% HCW) and the side effects of medication for poor adherence (8% CP vs. 83% CHW).

**Conclusions:**

Perspectives of CP and HCW likely reflect differing sociocultural and educational backgrounds. Health care workers must understand community perspectives on causes of suboptimal adherence as a first step toward effective intervention.

## Background

**Figure 1:**
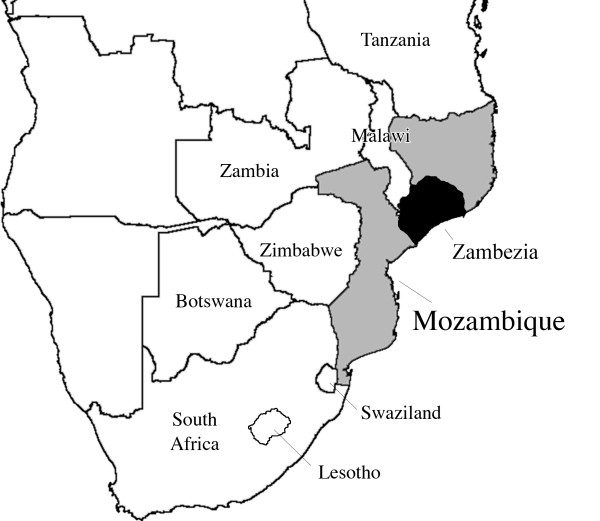
**Map of Southern Africa, highlighting Mozambique**.

Mozambique has one of the highest HIV infection rates in sub-Saharan Africa, with a national prevalence estimated between 11.5% and 16% [1-4]. Funding from international donors has allowed the Ministry of Health (MISAU) to expand HIV care and treatment services to rural communities [3-7], but only 24% of eligible patients in 2009 were accessing combination antiretroviral therapy (cART) [8]. Access to care and treatment is poorer in rural areas than in urban areas where, historically, educational and health care services were better established [1,9-12]. With funding from the U.S. President's Emergency Plan for AIDS Relief (PEPFAR), Friends in Global Health (FGH), which is affiliated with the Vanderbilt Institute for Global Health (VIGH), has partnered with MISAU to provide support for HIV care and treatment in clinics throughout Zambézia Province (Figure 1). Zambézia had an adult prevalence of 12.6% [4] in 2009. For cART to be effective, patients must adhere to their treatment regimens [13,14]. Poor adherence can lead to the emergence of drug resistance and eventually loss of immune function resulting in disease progression [15]. In rural Zambézia, the lost-to-follow-up rate among HIV-positive patients was ≈50% in 2008 (TDM, unpublished data).

Qualitative and quantitative studies have identified barriers to adherence, including: medication side effects, complexity of dosing schedules, low patient education levels, low socioeconomic status, stigma, transportation unavailability or cost, simply forgetting, and time away from employment [14,16,17]. Barriers and solutions to improve adherence in rural areas has been well documented in Haiti and Uganda [5,8,14,18], but only one study has been conducted in rural Mozambique [8]. In some studies, adherence rates in Africa have been documented to equal or exceed those in Western countries [19], while other studies have raised concern with current adherence rates and the measurement tools employed [20]. Explanations for high rates of adherence include use of social networks of HIV positive patients to encourage all members to take medications [21,22], non-adherent patients were excluded from treatment given limited availability [23], or that some cART programs provided effective, but unsustainable services and incentives for patients [24]. Currently, all HIV-positive patients in Zambézia Province receive free health care services, but nutritional supplements, transportation assistance, and financial incentives are rarely available. The rural location of our clinical sites, coupled with comparatively low levels of adherence, suggested the need to learn more about perceived barriers to patient adherence within our clinical setting.

## Methods

**Figure 2:**
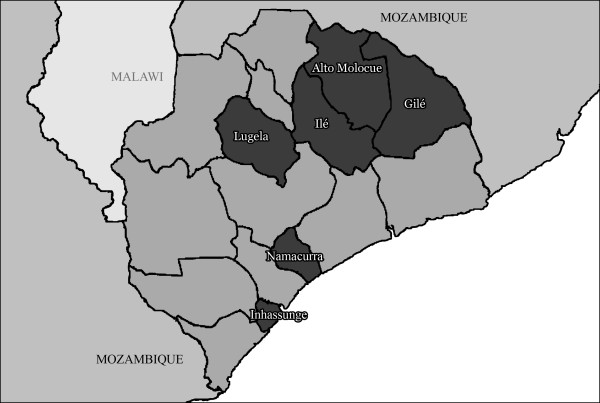
**Map of Zambézia Province, Mozambique**.

This study was carried out in six rural districts (Gilé, Ile, Namacurra, Inhassunge, Alto Molócuè, and Lugela) in Zambézia Province, Mozambique (Figure 2). The study was evaluated and approved by the Mozambican National Bioethics Committee and the Vanderbilt Institutional Review Board. We conducted three focus groups in each district to achieve saturation: one with women from the community, one with men from the community, and one with health care workers (HCW) who treat people living with HIV. Potential participants from the community were invited by a community leader to share their experiences and perceptions related to HIV and ART medication adherence, regardless of the given HIV serostatus of the participant. We requested the community leader obtain a reasonably diverse sample of the community by selecting participants from different families, neighborhoods, ages, and occupations. The community leader created a list of 30 people complete with basic demographic information for our team to review. Focus group leaders chose the final participants based on achieving a range of ages, families, and professions. Only one person per family was allowed to participate. All of those who were approached to participate agreed; all provided written consent. Community focus groups were conducted with same sex participants in each of the six districts. Community participants were asked "Why might people with HIV not take their medications every day, as instructed?" and "Why might people not seek or continue treatment for HIV?" We asked participants to respond to these questions only if they knew someone close with them who had been using ART of if they themselves were on medication. We believe this was useful as participants could respond about specific issues without having to claim the experience as their own. While the last question requested information about seeking and continuing treatment for HIV, only responses related to continuation of HIV treatment were analyzed and included in this manuscript.

In addition to community groups, focus groups of 5 to 11 health care workers (mixed gendered) were conducted in each of the 6 districts. Health care worker participants were asked "What do you think are the biggest barriers to ART adherence for your patients?" Health care workers were encouraged to talk about both formal medical adherence (how many pills taken per day) and about why patients may stop taking their medication all together. We recognize that the questions for community and HCW were not exactly the same, but as education levels were low among community participants, we felt these questions would get at the important question of why people did not always take medicine or would interrupt their HIV care.

The focus groups were conducted by trained moderators (all had previous experience conducting multiple focus groups in Mozambique) who spoke Portuguese, Chuabo and Lowme. All community focus groups were conducted in the preferred local language. Participants were asked a variety of questions regarding perceptions of HIV illness as well as perceived barriers to ART adherence. Sessions were audio taped and translated/transcribed into Portuguese and English. Transcriptions were reviewed by three members of the team to ensure that the spirit of the responses was preserved in the translation. Data were analyzed using content analysis in which no preconceived categories were established and researchers independently created categories as they coded the data into important themes. Data were coded by hand, without using a computer program. Two authors (KG and CA) coded the data separately and compared results; only a single interpretive category discrepancy was discovered. This was discussed and was reconciled by collapsing two data categories into one.

## Results

### Characteristics of participants

Of the 76 women and 88 men who participated in our focus groups, 124 were volunteers from the community (60 women, 64 men) and 40 were health care workers (16 women, 24 men). No one refused to participate in the focus groups. Community participants had a mean age of 35 years, had low levels of education (mean of 4 years), and primarily worked as subsistence farmers (73%). Health care workers were younger than the community group (mean of 30 years) and were better educated (average of 11 years; most with a post secondary degree); 55% were in health care services (pharmacist, laboratory technician, medical technician, information technician, nurse) and 45% were in social services (social assistant, peer educator, counselor).

### Economic Barriers to Adherence

Economic obstacles to ART adherence were noted by all groups and included financial barriers to procuring food and transportation. Identified by 14 of 18 (78%) focus groups, the inability to afford quality or sufficient food was widely reported as a barrier to ART adherence. Patients who begin ART regimens often experience an increased appetite, resulting in "unrelenting hunger" [25], which in turn lead to anxiety about lack of food, a factor in subsequent non-adherence, in the view of the focus group members. A man in the district of Alto Molócuè said, "The problem here has to do with economic conditions. The problem with food is huge. It's not possible to eat *caracata *[boiled flour meal] with dry salty fish. Problems with illness are aggravated because of the lack of food." One HCW agreed, stating, "Nutrition is complicated. When projects that give food to people on ART stop, the people stop returning."

Noted by 12 of 18 groups (67%), inadequate transportation was an important barrier to cART adherence. Some participants may have the financial means, but are impeded by the poor quality of roads and unreliability of public transportation. A man from the district of Lugela noted that, "Others don't get treated due to a lack of transportation. Those who live far away have difficulty accessing treatment. The roads and bridges aren't good." A HCW from Alto Molócuè agreed, "Our geographic distribution is a factor. People who live far away abandon treatment." As cART patients in Mozambique are required to return to the clinic each month to receive their medications, it was noted that this might create a significant strain on the financial resources of some patients.

### Patient-Provider Challenges

In addition to economic barriers, poor relationships and conflicts with health worker staff may also deter patient adherence. Men in particular focused on the lack of confidentiality and poor treatment by hospital staff. A man from Ile said,

They should put a doctor here because the people here don't work at all. The other day a woman died there in the hospital because they didn't treat her. People prefer to die with traditional medicine because the hospital doesn't treat them

Another added,

If [a patient] asks [a pharmacist] to repeat what they said because [the patient] didn't understand, [the pharmacist] is insulted. [The pharmacist will] say, 'sir, you should study at a school.' And they say this in the presence of many women. The women sit there and laugh."

The clinical sites at all of these locations are small, and confidentiality is difficult to maintain. In some clinics, there are particular offices used only for the testing and treatment of HIV-positive patients, creating inadvertent patient disclosure. Pharmacy windows are crowded. The likelihood of a patient receiving confidential service in this context is very limited.

HCW also note the challenges of creating good relationships and communicating information about HIV to their patients. Health care workers suggest language barriers and problems with patients being overwhelmed with information. A HCW from Lugela noted, "Some people don't understand what we say, but always respond affirmatively by nodding their heads." In large part, language is the barrier to effective communication. This was noted by many in the community, including a man from Namacurra who said,

They explain things in Portuguese in the hospital. The only things we know how to say are head and belly [in Portuguese]. Sometimes when we have chest pain, we say we have belly pain because we don't know how to say "chest." When we have ear pain, we say "head" because we don't know how to say "ear" and the nurse gives us a prescription that functions for my complaint.

The language barrier creates a challenge in ensuring that patients truly understand the importance of ART adherence or how to take their medication properly. A doctor from Gile explained,

The counselor has to do a study of the person, if we can or can't talk about the subject, or postpone it. We shouldn't fill the person with information because they aren't going to understand everything we tell them.

Health care workers implicated ART side effects as being one of the most important barriers to adherence, but this was not echoed by community participants (8% of community focus groups vs. 83% of health care worker focus groups). A HCW in Namacurra gave an example of how side effects have affected patients:

There have been 2 or 3 cases of people whose skin looked burned ... These patients have a tendency to tell us that the medicines that the hospital gives are not good. We need to re-explain that side effects are possible and it's important not to abandon treatment.

Men living in Alto Molócuè, the only larger town in the study, were the only community focus group to present this as a barrier. One man said,

The nurses don't inform [patients] of the types of reactions they might have. When the medical technician gives the prescription and afterward explains what the side effects are, the sick person won't stop taking the medicine. I don't know if there is a person here who has ever been told of side effects.

### Social Barriers to Adherence

Four barriers to adherence that exist outside of the clinical arena were noted by focus group participants. These included the practice of saving medication, the belief that medication should be curative, the use of traditional healers, and the existence of stigma surrounding HIV infection. The first three are cultural practices that precede the coming of the HIV epidemic, while the forth (stigma) is linked specifically to HIV infection and AIDS but is exacerbated by accepted gender norms and expectations.

Eight of 12 (66%) community groups referred to the practice of saving medicine to give to others. One woman from Ile stated, "When we receive 4 pills and our children are sick, too, we divide them: 2 for me and 2 for them. You see this often in our community." Pills are also shared between other family members and friends, creating a perceived social network of support. Others save their medication for their own use at a later time, in case the illness recurs. A man in the district of Lugela explains,

You can go to the hospital and buy your medicine and return to your house and begin to take it. Two days later you feel well and because you know you don't have more money, you stop taking it so that when you're sick the next time, you can take the same medication even though you don't know if it will cure you or make you worse.

Many people in the community believed that if a medicine is good, it should be curative. None of the health care worker focus groups voiced concern about the effectiveness of ART, but 9 of 12 (75%) community focus groups believed this to be a barrier. The concept of chronic diseases that require a lifetime of treatment is a relatively new idea in rural Mozambique, and not well understood or widely accepted. "People have the idea that medicines cure in the first 3 days," said a man from Alto Molócuè, "I'll take the medicine today, tomorrow, and the day after, and if I'm not cured, I have to abandon treatment to find other medicines." Another woman in Gile noted,

"People don't go to the hospital because they know that they won't be cured, but are only given information cards ... I [may] go to the hospital feeling well but leave ill. But why, since I was feeling well? Therefore, it's preferable to stay home, with or without AIDS."

The juxtaposition of having an illness even if a person has no symptoms has no correlate in local understanding of disease, given that there is no tradition of chronic disease control and management (e.g., for hypertension or rheumatoid arthritis), making chronic cART for HIV a difficult concept for many to understand.

Only 5 of 12 (42%) community groups indicated that patients' preferences for traditional healers were a barrier to ART adherence, whereas all 6 (100%) of the health care provider focus groups cited this to be a barrier. A health care worker from Namacurra said,

There are many cases when patients prefer traditional treatments [to their ART]... Much later, the patient stops that treatment and returns to the health center, but many times they are already in the final phases and they come to die here."

A community member from Namacurra expressed one reason why medication is sought from a traditional healer,

First we go to the hospital, and if we're not cured, we go to the traditional healer to try because sometimes traditional medicine is very strong and good because it hasn't been modified chemically.

Participants suggested patients' often preferred to seek care with a traditional healer because of the respect given to patient's concerns and their ability to effectively communicate with the healer in their native language.

The impact of stigma on adherence for many illnesses is well documented in the literature [26-29]. Noted in 14 of 18 (78%) groups, stigma directed toward those infected and the resulting fear of disclosure felt by those who test positive has a negative impact on adherence to ART. This was a particular challenge for men and women in sexual relationships. A medical technician in the district of Gilé stated,

Well known people abandon treatment because they don't want anyone to know they are HIV-positive. This is a big problem because I see them dating people I know, who were not HIV-positive, but now are.

Both men and women have the additional challenge of engaging in sexual relationships while maintaining their health and that of their partners. A HCW in Inhassunge noted, "Women are afraid to tell their husbands. When they have to hide their illness in order to avoid divorce, they often abandon treatment." The fear of losing a partner was so strong that often people would risk their own health to avoid disclosure. For women, disclosing their health status could lead to divorce, which could result in the loss of their children and their financial stability, as men could force them to leave the family home.

## Discussion

Barriers to adherence perceived by focus groups, both community participants and health care workers, included: the inability to afford food in sufficient quantity or quality, a lack of transportation availability, and stigma/shame. These barriers have been cited in studies in other low income countries and may be especially acute in a rural, impoverished setting [10,16,25,30,31]. Several factors perceived as barriers by community participants appeared less important to health care workers and vice versa. While our study was not designed to compare these two groups with statistical rigor, the differences in their perspectives provide insight into potential interventions that may bolster adherence. Several interventions can be developed on the basis of information gathered during our study.

1. *Provide food supplementation to patients enrolled in care*. The issue of food insecurity is particularly challenging in Zambézia, as the province has high background rates of moderate and severe malnutrition among young children [32]. In 2008, 46% of children in Zambézia Province suffered from moderate or severe malnutrition, suggesting that even when illness is not a factor, acquiring adequate and nutritious food in the province is a challenge [33]. Our PEPFAR program in Zambézia does not routinely provide nutritional supplements to HIV positive patients (as per PEPFAR and MISAU guidance), but clinicians and community members agree that adding food supplement programs would be a means of improving patient adherence to medication.

2. *Improve patient/provider relationships*. Community participants complained about the lack of confidentiality and poor treatment by hospital staff. Men appear to be particularly sensitive to degrading comments or treatment by HCW. Studies from Tanzania, Zimbabwe, and South Africa have documented poor relationships between clinicians and patients have negative impact on drug adherence [34-36]. Creating effective communicating between patients and clinicians, who are from very different social and/or ethnic background is challenging [37]. Cultural training and solid communication skills are necessary [38]. To improve service quality, we have begun training in effective communication skills and have created screened areas around pharmacy counters to provide more privacy for patients.

3. *Integrate traditional healers and religious healers into the medical system*. The treatment activities of traditional practitioners have come under scrutiny in recent years [39-47]. Our previous study conducted in Zambézia suggested that a majority of the population has sought care from a healer, but often seeks care from a clinical site first [48]. Participants who spoke about traditional healers as barriers to clinical care primarily shifted the "blame" of their using traditional medicine onto clinical workers who were ineffectual. Many state that they only seek care from a healer if they were told nothing could be done for them at the clinic. Use of complementary and alternative medicines has been found to delay patient initiation of ART in a recent Gabon study [49], while others have noted an association of alternative medicine use with poor ART adherence [50]. In Zambézia, we have begun training traditional healers about HIV diagnosis, treatment and co-infections, and we have established a system of patient referrals. We are hopeful that better coordination with traditional healers may forge a therapeutic alliance for better overall HIV care, but we do not yet know if this will be achievable

4. *Engage 'expert' patients to mentor those initiating ART*. Culturally specific understanding of illness and treatment may be more effectively addressed by someone who shares the same background. Given the historical absence of any chronic disease management infrastructure in rural Mozambique, we were not surprised that the expressed belief that "good" medicine will "cure within a few days" was common within our community focus groups. This view of illness as acute and transient has been shown to contribute to poor adherence in other communities [28].

In addition to traditional understanding of effective treatment, the sharing of ARVs was cited by community participants as a barrier to adherence. This phenomenon has been documented elsewhere [51-53]. Patients may have resorted to sharing medication during "stock-outs" that have occurred, especially early in ART roll-out. However, we hypothesize, based on comments from participants, that sharing medication may be more strongly related to the establishment of social networks among community members. With high levels of poverty in the province, sharing medicine of any sort may provide a social safety net for rural poor, as many medications are costly. While ART is provided free of charge, the travel cost and time associated with traveling to clinical sites may induce some to borrow medication from friends or family members. Given the prevalent view that illnesses have a spiritual origin, rather than an infectious one, we speculate that rigid adherence is a goal of health care workers more often than patients [48]. To counter these beliefs we believe expert patients, can effectively teach new patients skills to successfully adhere to their medication as has been found in Mexico [54].

We believe our study provides new insights into ART adherence that can inform programmatic changes within rural Mozambique. The strengths of our study are the broader insights provided by the complementary focus groups, both in the community and among HCW. Conducting focus groups with men, women and HCW in each community allowed us to effectively reach a diverse group of participants. A limitation of our findings is that focus groups are known to elicit more views of verbal participants than shy ones. While we attempted to sample for diversity, our participants were not selected randomly and were chosen by community leaders. As HIV status was not a factor in participation, it is likely that many were not HIV positive. Some participant's spoke of their own experiences with HIV, while others recounted stories experienced by their family and friends. In addition, translation from local languages to Portuguese (and subsequently to English) further complicates analysis. The challenge of cultural understanding can be difficult in rural areas where only local languages are understood, though research leadership from a medical anthropologist (CMA) was useful. While we have made all attempts to maintain the integrity of our data, it is possible that some words or phrases were incorrectly translated, though we believe the substantive viewpoints were correctly inferred.

## Conclusion

Focus group discussions highlighted differing perceptions of barriers to adherence among community participants and health care workers. Economic and logistical barriers, including a lack of food and transportation, were almost universally noted by both groups. Cultural barriers were also noted, including health care worker rudeness and disrespect, sharing of antiretroviral drugs with family and friends, and the view of illness as acute and transient. Hence, both structural changes to programs and culturally sensitive education efforts are needed to improve adherence. Effective partnerships with traditional healers would be beneficial given the frequency with which their services are sought for HIV and non-HIV related conditions alike [48,55]. Promoting better dialogue between patients and providers can probe patient fears and concerns and provide insight into potential challenges to optimal patient adherence. In the absence of resources necessary to provide transportation and food subsidies to patients on ART or employ armies of community health workers facilitating directly observed ART administration, ART programs in rural Africa face daunting challenges to ensure adequate adherence.

## Competing interests

The authors declare that they have no competing interests.

## Authors' contributions

The review protocol was developed by all authors. KG conducted the focus group interviews. KG and CMA coded and analyzed the data. All authors contributed to the writing of this paper. All authors read and approved the final manuscript.

## Pre-publication history

The pre-publication history for this paper can be accessed here:

http://www.biomedcentral.com/1471-2458/11/650/prepub
